# Detection of titin fragments in urine in response to exercise-induced muscle damage

**DOI:** 10.1371/journal.pone.0181623

**Published:** 2017-07-20

**Authors:** Kazue Kanda, Jun Sakuma, Takayuki Akimoto, Yasuo Kawakami, Katsuhiko Suzuki

**Affiliations:** 1 Institute for Nanoscience & Nanotechnology, Waseda University, Shinjyuku, Tokyo, Japan; 2 Department of Health Science, Musashigaoka Junior College, Yoshimityo, Saitama, Japan; 3 Division of Regenerative Medical Engineering, Centre for Disease Biology and Integrative Medicine, Graduate School of Medicine, The University of Tokyo, Bunkyo, Tokyo, Japan; 4 Faculty of Sport Sciences, Waseda University, Tokorozawa, Saitama, Japan; Biomedical Research Foundation, Academy of Athens, GREECE

## Abstract

Many studies have attempted to determine the associations between blood biomarkers and exercise-induced muscle damage. However, poor correlations between the changes in biomarker levels and the magnitude of muscle symptoms have been reported. Recent advances in proteomic tools offer a strategy for the comprehensive analysis of protein expression, which can be used to identify biomarkers. Here, we used a proteomic analysis to identify urinary proteins that appear in response to a calf-raise exercise, including repetitive eccentric muscle contractions, and found that a titin (also known as connectin) N-terminal fragment molecule appears in the urine after eccentric exercise. We measured the titin fragment in urine samples from nine individuals before and after eccentric exercise using a newly-established enzyme-linked immunosorbent assay and found that the titin fragment excretion rate increased 96 h after the exercise (5.1 to 77.6 pg/min, p <0.01). The changes in the titin fragment excretion rate were correlated strongly with blood markers of muscle damage and with muscle symptoms. These findings suggest that the urinary titin fragment is potentially a noninvasive biomarker of muscle damage.

## Introduction

Muscle damage is readily induced by eccentric exercise [1, 2], causing skeletal myofiber disruption, inflammatory cell infiltration, and muscle soreness [3, 4], similar to the pathology of myopathy. The direct assessment of muscle damage involves a morphological evaluation of the skeletal muscle [5, 6, 7]. However, it is sometimes difficult to find the disrupted tissue because eccentric muscle contraction induces sporadic muscle disruption. Moreover, researchers generally hesitate to take muscle biopsy due to its invasiveness, so that researchers have been searching for noninvasive ways to assess muscle disruption for use in both the clinical and laboratory contexts.

The activities of muscle-derived enzymes in the blood, such as creatine kinase (CK), lactate dehydrogenase (LDH), and the myocellular protein myoglobin (Mb), which leak into the circulation from damaged muscle, have been used as indirect markers of muscle damage [8, 9, 10]. Delayed-onset muscle soreness (DOMS) and changes in the range of motion (ROM) have also been used as indicators of muscle damage [1, 11]. However, poor correlations have been reported between the changes in the concentrations of muscle damage markers and the magnitude of muscle symptoms after eccentric exercise [12]. It has also been reported that the serum levels of these biomarkers depend on sex, muscle mass, and the intensity and duration of exercise [2]. There is also remarkable interindividual variation in the degree to which serum enzyme activities increase with exercise [2, 13]. Therefore, there are very few noninvasive and sensitive biomarkers that accurately reflect exercise-induced muscle damage.

Recent advances in proteomic tools offer a strategy for the comprehensive analysis of protein expression, which can be applied to the search for biomarkers. In fact, many proteomic studies have already been used to identify biomarkers among serum, urinary, and salivary proteins for the early diagnosis of various diseases, including cancer [14] and Alzheimer’s disease [15]. In the context of exercise-induced muscle damage, Malm et al. reported that the expression of several Z-band-related proteins was detected in the serum after eccentric exercise [16]. Sietsema et al. reported that alpha 1-antichymotrypsin and C-1 protease inhibitor peptides increased earlier than CK after exercise, and suggested these proteins as new biomarkers of muscle injury [17]. However, to date, no study has used proteomics to analyze urinary proteins after eccentric exercise.

In this study, we performed a comprehensive proteomic analysis to identify urinary proteins that are responsive to acute eccentric exercise. We found that an N-terminal fragment of titin (also known as connectin) is detectable in urine after eccentric exercise. We then established a quantitative enzyme-linked immunosorbent assay (ELISA) to measure the urinary titin fragment, and evaluated the utility of the urinary titin fragment as a biomarker of exercise-induced muscle damage.

## Materials and methods

### Subjects

Nine healthy males participated in the original investigation [18]. In the present study, urine samples from those subjects were analyzed during the experimental period. The mean (± SD) characteristics of the subjects were as follows: age 24.8 ± 1.3 years, body mass 62.3 ± 6.3 kg, and height 1.72 ± 0.05 m. The subjects were instructed to maintain their usual daily schedules during the experiment. The study protocol was approved by the Ethics Committee of Waseda University, Japan, and the subjects gave their written informed consent.

### Experimental design

The subjects performed a calf-raise exercise, including repetitive eccentric muscle contractions, with their right leg on a force plate, as described previously [18]. Briefly, each subject rested on an exercise device specially designed for ankle plantar flexion, with the knee joint extended and the metatarsal bone resting on a stool. The slope of the backrest was 30°, so that the exercise load corresponded to approximately half of the subject’s weight (exercise load = body mass × sin 30°). With their right leg, subjects performed single-leg ankle plantar flexion exercise consisting of 10 sets of 40 repetitions with a 3-min rest between sets. The ROM of the ankle joint during the exercise was maintained between 20° (dorsiflexion position) and 15° (plantar flexion position) using an electronic goniometer (SG110/A, Biometrics, Newport, UK) with its ends attached to the distal-lateral part of the fibula and the lateral part of the foot. Each subject received visual feedback on his ankle joint ROM during the exercise via display of the joint ROM value on a personal computer. The exercise was performed in accordance with the rhythm of an electrical metronome at a speed of 60 counts/min; ankle dorsiflexion and plantar flexion were alternated and repeated every 1 s. All subjects completed a total of 400 repetitions of ankle plantar flexion. Delayed-onset muscle soreness (DOMS) was rated with a visual analogue scale (VAS): a 100-mm line with “no pain” at one end and “extremely sore” at the other. The tenderness of the exercised muscle correlative to DOMS was assessed using the FP meter (SN-402, Navis, Japan) at 1 kg. The point of measurement was the middle point of medial gastrocnemius [9]. Blood and urine samples were collected before and 2, 4, 24, 48, 72, and 96 h after exercise. Serum Mb measurements were made as described previously [18]. Serum CK, LDH, and aldolase (ALD) were measured as described by Kanda et al. [9, 18].

### Human urine protein concentrates

The urine samples were centrifuged immediately at 1000 × g for 10 min to remove the sediment, and the supernatants were stored at –80°C for later analysis. The 50 ml samples were transferred to Amicon^®^ Ultra 4 Centrifugal Filter membrane concentrators (molecular weight cut-off 3K; cat. no. UFC800324, Millipore, Billerica, MA, USA) and centrifuged at 7000 × g for 6 h to reduce their volumes to 20 ml, after which 20 ml of lysis buffer (4% (w/v) CHAPS, 2 M thiourea, 8 M urea, 10 mM Tris-HCl [pH 8.8]) was added to the samples. The amounts of protein in the urine concentrates were measured with the BCA assay kit (Pierce, Rockford, IL, USA) and frozen at –80°C for later analysis.

### Gel electrophoresis and imaging

Immobilized pH gradient (IPG) strips (pH 3–10, 24 cm) were rehydrated and the prepared samples were applied with cup loading. Isoelectric focusing was performed with a Multiphor™ II electrophoresis unit (Amersham Biosciences, Little Chalfont, Bucks, UK) for 54 kVh at 20°C in the dark [19, 20]. The strips were equilibrated for 10 min in buffer (50 mM Tris-HCl [pH 8.8], 6 M urea, 30% [v/v] glycerol, 1% [w/v] sodium dodecyl sulfate [SDS]) containing 65 mM dithiothreitol, and then for 10 min in the same buffer containing 240 mM iodoacetamide. The equilibrated IPG strips were transferred onto 24 cm × 20 cm, 12% T, 7.5% C polyacrylamide gels made between low-fluorescence glass plates. The strips were overlain with 0.5% (w/v) low-melting-point agarose in buffer (25 mM Tris-base, 0.1% SDS, 192 mM glycine) containing 0.1% bromophenol blue. The gels were run in the Ettan DALT Twelve Electrophoresis System (Amersham Biosciences) at 2 W/gel at 20°C, until the dye fronts had run off the bottom of the gels. The two-dimensional (2-D) gels between low-fluorescence glass plates were scanned directly with a Typhoon 9400 imager (Amersham Biosciences). Normalization of the three Cy™ dyes was accomplished by adjusting the maximum pixel values to 55.000 counts by changing the photomultiplier tube voltage. The images generated were exported as tagged images (Amersham Biosciences).

### Image analysis

The differential in-gel analysis with DeCyder™ was used to merge the Cy2, Cy3, and Cy5 images for each gel, and to detect the spot boundaries to calculate the normalized spot volumes/protein abundance. At this stage, features resulting from nonprotein sources (e.g., dust particles, streaks) and faint spots (e.g., spot areas ≤ 300, spot volumes ≤ 10.000) were filtered out. The analysis was used to calculate the abundance differences between samples run on the same gel. The biological variation analysis (BVA) of DeCyder™ was then used to match all pairwise image comparisons from the DIA for a comparative cross-gel statistical analysis. Comparison of the normalized Cy3 and Cy5 spot volumes with the corresponding Cy2 standard spot volumes within each gel gave the standardized abundance. This value was compared across all gels for each matched spot. All analyzed gels were matched to one “master gel” to assign the same number to the same protein spot. The master gel image was obtained from the pooled sample derived from all urine samples.

### In-gel digestion and peptide extraction

Gel electrophoresis for mass spectrometry (MS) analysis was performed with the procedures described above (Gel electrophoresis and imaging). After electrophoresis, the gel was fixed in 10% (v/v) methanol:7% (v/v) acetic acid and stained with Sypro^®^ Ruby. This gel for MS analysis was then matched to the master gel for expression analysis with the BVA software. Spots of interest were excised from the two-dimensional (2D) gels using an automated spot picker (Amersham Biosciences), according to the manufacturer’s instructions. The spots were collected in 200 mL of water in 96-well plates. The recovered gel pieces were washed with aqueous 50 mM ammonium bicarbonate and acetonitrile (ACN), and then incubated with 12.5 ng/mL trypsin (Promega, Southampton, UK) at 30°C for 15 h. The peptides generated were eluted with 50 mM ammonium bicarbonate followed by 10% (v/v) formic acid and ACN. The combined fractions were dried in a Speedvac and dissolved in 0.1% (v/v) formic acid.

### MS analysis

The MS analysis was performed with DIGE [21, 22]. A high-performance liquid chromatography (HPLC) apparatus (CapLC, Waters, Milford, MA, USA) was coupled to a quadrupole-time of flight MicroMass spectrometer (Micromass, Manchester, UK). Instrument operation and data acquisition and analysis were performed with the MassLynx 3.2 software (Micromass). The tryptic peptides were concentrated and desalted on a 300 mm i.d./5 mm C18 PepMap column (LC Packings, San Francisco, CA, USA). The eluted peptide was analyzed with MS/MS sequencing with an automated MS-to-MS/MS switching protocol. The precursor ion masses were determined online over an m/z range of 400–1600 amu in the positive charge detection mode, with a cone voltage of 50 V. The cone voltage, extraction voltage, microchannel plate detector voltage, and collision energy were optimized before the measurement of the samples. A database search was performed with Mascot Deamon (Matrix Science, London, UK) [23, 24]. The generated pkl files were submitted to Swiss-Prot (release 47.4) and NCBInr (14-Jul-2005). The search parameters were as follows: fixed modifications, carbamidomethyl; variable modifications, oxidation (M); missed cleavages, up to 1; monoisotopic; peptide tolerance, 1.0 Da; MS/MS tolerance, 0.5 Da. The ion score cut-off was set to 20. The automatically identified proteins were manually checked individually to remove any redundancy.

### Immunoblotting analysis

The collected urine was mixed with complete protein-loading buffer containing 50 mM Tris-HCl (pH 6.8), 1% SDS, 10% glycerol, 20 mM dithiothreitol, 127 mM 2-mercaptoethanol, and 0.01% bromophenol blue, supplemented with protease inhibitors (Roche) and phosphatase inhibitors (Sigma-Aldrich, St. Louis, MO, USA). The urine samples were transferred to microfuge tubes, heated for 5 min at 100°C, and centrifuged in a microfuge for 5 min at 12,000 × g at room temperature. The urine samples were then loaded onto 7.5%–15% gels (depending on the molecular weight of the protein) for SDS–polyacrylamide gel electrophoresis (PAGE), transferred to a nitrocellulose membrane, and immunodetected with an enhanced chemiluminescence kit (ECL prime, Amersham) using the LAS-3000 Imaging System (Fuji Film, Tokyo, Japan), as described previously [25]. Antibodies directed against the titin fragment were used for the immunoblotting analysis: anti-TTN antibodies clone 7D3, clone 2B3, and clone 2F12 (Abnova, Taipei, Taiwan), and rabbit anti-titin polyclonal antibody (CMD1G; Bioss, Boston, MA, USA). The secondary antibodies used were horseradish peroxidase (HRP)-conjugated sheep anti-mouse IgG antibody (NA931; Amersham) and HRP-conjugated goat anti-rabbit IgG antibody (Amersham). The proteins were quantified with the ImageJ software (NIH, Bethesda, MD, USA).

### ELISA

The concentrations of the titin fragment were quantified with an ELISA. Briefly, a 96-well microtiter plate (Immulon II, Dynex Technologies, Chantilly, VA, USA) was coated with an anti-human titin monoclonal antibody (clone 7D3; Abnova) and incubated overnight at 4°C. The wells were blocked with the addition of 250 μl of phosphate-buffered saline (PBS) containing 1% bovine serum albumin (BSA; Sigma, St. Louis, MO, USA) for 2 h. The urine samples were thawed, centrifuged at 10,000 rpm for 5 min, and diluted (1/3) with PBS containing 1% BSA. An aliquot (100 μl) of each sample was added to one well and incubated for 1 h. Known concentrations of the N-terminal fragment of recombinant human titin (amino acids 1–111, Q01, Abnova) were also plated to establish standard values. The plate was washed with PBS–Tween, and another anti-human titin monoclonal antibody (clone 2B3, Abnova) was added to the wells, which were incubated for 1 h. After the plate was washed with PBS–Tween, a rat anti-mouse IgG1 antibody conjugated with HRP (ab99603, Abcam) was added to the wells and incubated for 1 h. After the wells were washed, substrate solution was added to them and the intensity of the color produced after 15 min was measured with a microplate reader (ARVO MX; PerkinElmer, Waltham, MA, USA) at 490 nm. All samples were assayed in duplicate and the average absorbance was used to represent the fragment concentration. A regression analysis of the relationship between the standard titin fragment concentration and absorbance was used to interpolate the concentrations of the titin fragment in the samples. To avoid interassay variability, all samples from each subject were assayed on the same plate. Then, the titin fragment excretion rate was calculated with urine volume and elapsed time.

### Statistical analysis

The data were analyzed with one-way analysis of variance. When significant time effects were evident, multiple comparisons were analyzed with the Bonferroni adjustment. Associations between data were analyzed with Pearson’s correlation coefficient (r). Statistical significance was set at p < 0.05, and the data are presented as means ± standard error (SE).

## Results

### Muscle strength

As shown in Table 1, the torque of the ankle joint decreased significantly after eccentric exercise (p < 0.01 and p < 0.05).

**Table 1:** Changes in the torque of the ankle joint after the eccentric exercise.

	Pre	Post	24h	48h	72h	96h
Means (Nm)	152.6	90.9 **	131.5 *	134.7	135.3	147.1
SD	32.3	21.4	30.2	36	34.5	33.8

*: Significant changes at p<0.05.

**: Significant changes at p<0.01.

### Proteomic analysis

**Fig 1:**
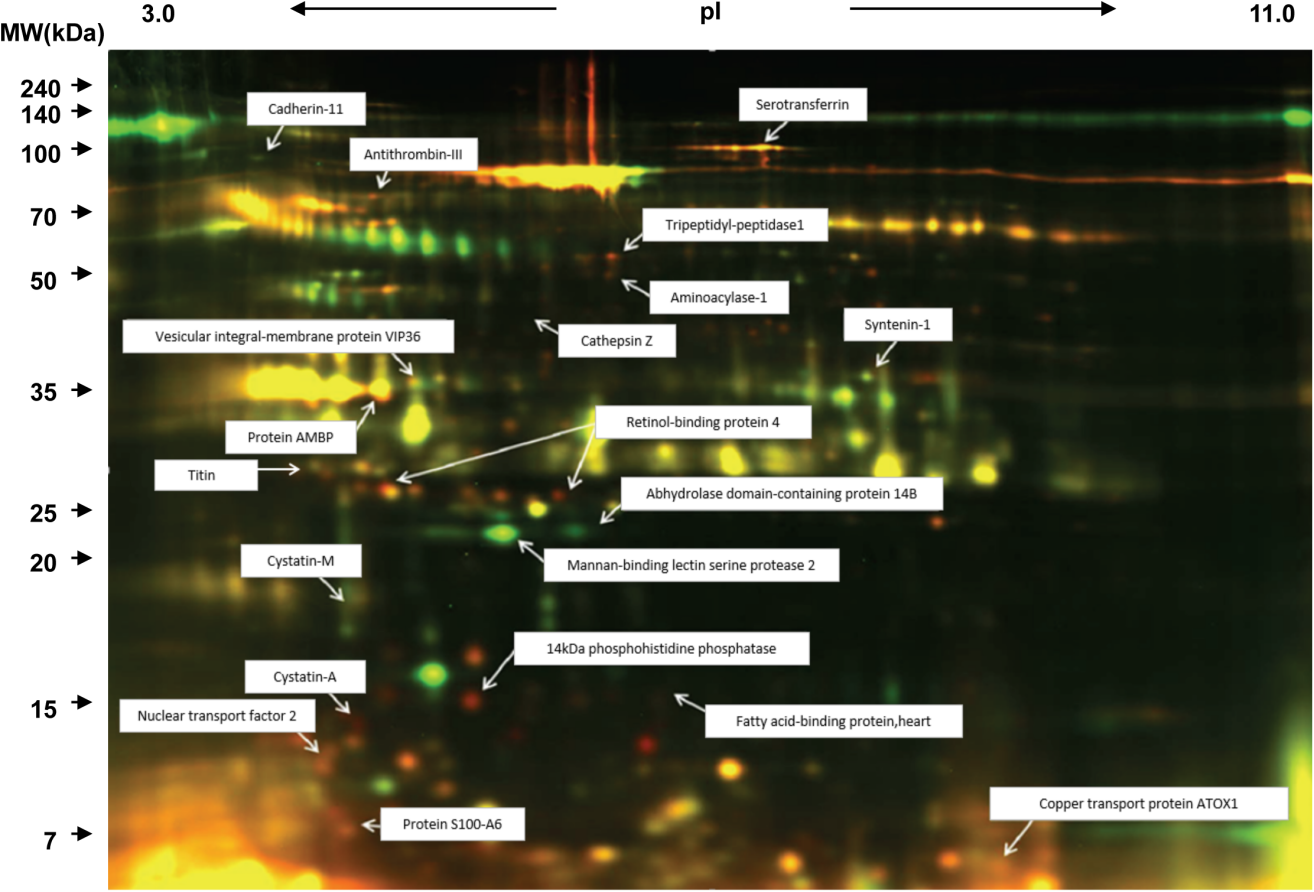
Typical 2D-DIGE images before and after eccentric exercise. Urine samples were labeled with Cy3 (before) or Cy5 (immediately after). Red spots reflect increased protein expression after exercise.

A typical 2D differential in-gel electrophoresis (2D-DIGE) image is shown in Fig 1. From the 2D-DIGE gel patterns, specified for each protein in S1 Table, we identified approximately 99 proteins (156 spots) as uniquely expressed after eccentric exercise.

### Titin N-terminal fragment

**Fig 2:**
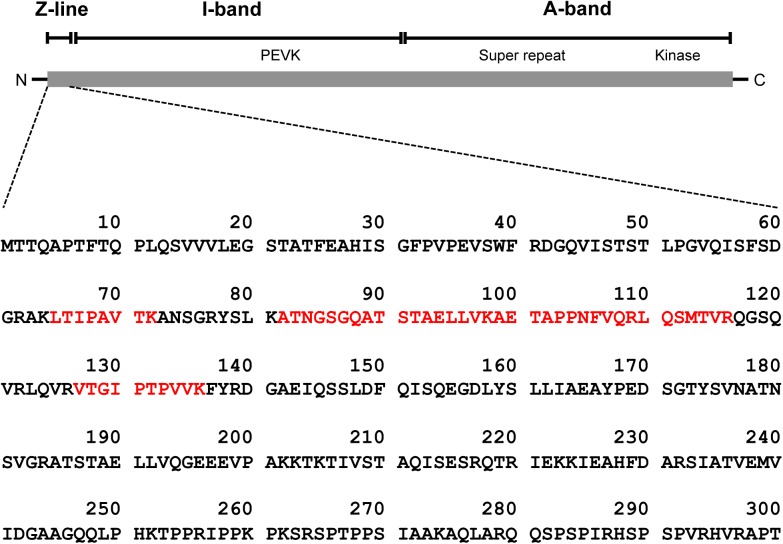
A schematic diagram of human titin. The N-terminal titin fragment was identified with the protein database search engine MASCOT. The sequences in red text were detected with LC–MS/MS.

**Fig 3:**
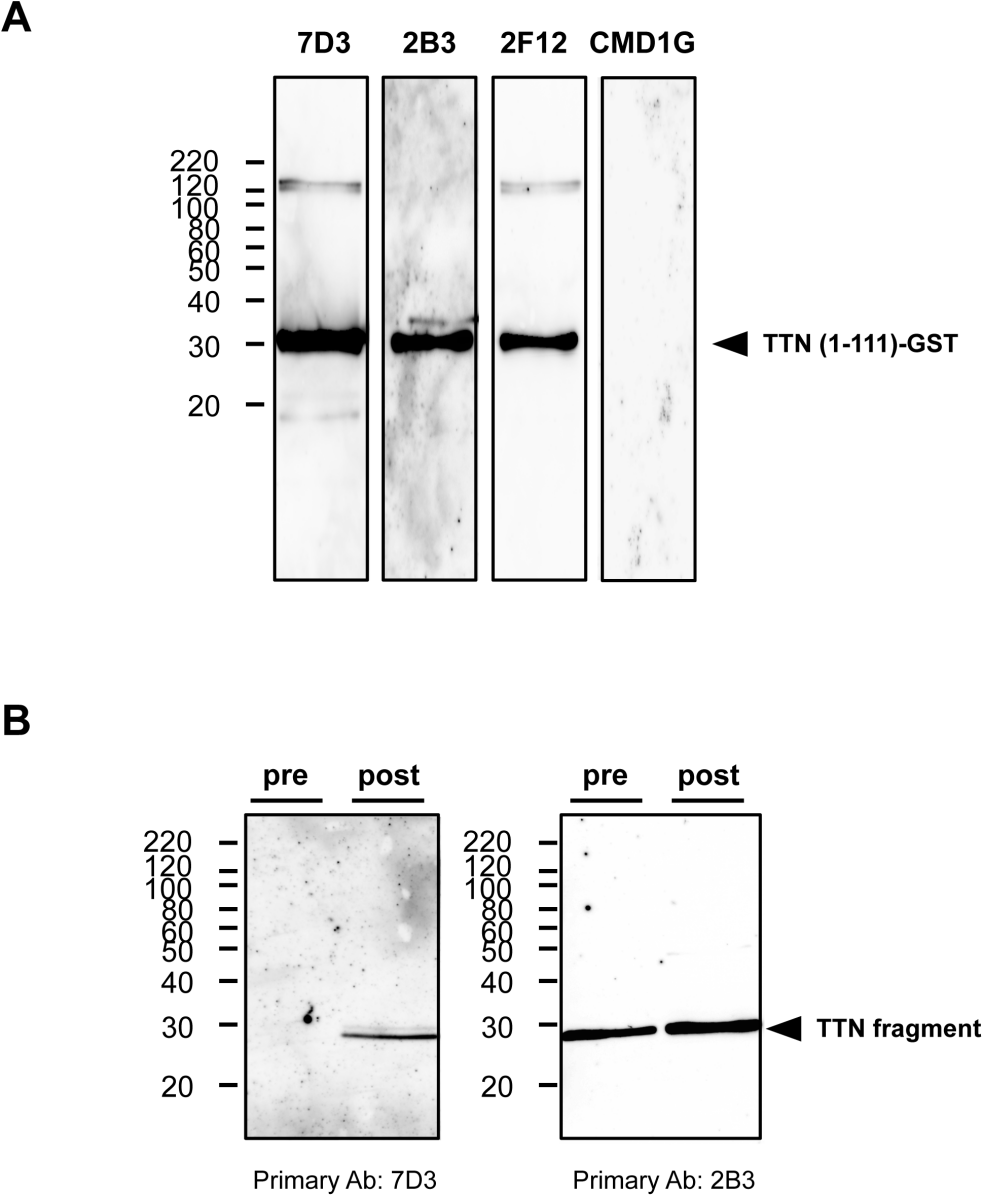
Reactivities of antibodies against the N-terminal fragment of human titin. (A) To identify antibodies that react with the N-terminal fragment of human titin, a recombinant human titin fragment (amino acids 1–111) was loaded onto an SDS-PAGE gel, and the antibody reactivities were evaluated with immunoblotting using an appropriate secondary antibody. Anti-human titin antibody clones 7D3 (1:1000), 2B3 (1:1000), and 2F12 (1:1000) detected the recombinant titin protein. Anti-human titin antibody CMD1G (1:500) did not detect the protein. The arrowhead indicates the GST-tagged recombinant human titin (amino acids 1–111). (B) To confirm the reactivities of the antibodies, urine samples (pre and post: before and 96 hours after the eccentric exercise, respectively) were loaded onto an SDS-PAGE gel, and the antibody reactivities were evaluated with immunoblotting using an appropriate secondary antibody. The arrowhead indicates the N-terminal fragment of human titin.

We identified a titin N-terminal fragment (molecular weight, 28 kDa) as one of the 99 proteins displaying altered expression after eccentric exercise (S1 Table and Fig 2). As shown in Fig 3A, primary mouse monoclonal antibodies specific for titin, clone 2B3 (1:1000; Abnova) and clone 7D3 (1:1000; Abnova), detected a glutathione S-transferase (GST)-tagged recombinant human titin (amino acids 1–111; H00007273-Q01, Abnova), whereas clone 2F12 (1:1000; Abnova) gave weaker signals. Next, we determined whether these antibodies detected the titin fragment in human urine samples. As shown in Fig 3B, clones 7D3 produced signals in the urine samples obtained after eccentric exercise but not in the preexercise samples. The monoclonal antibody, clone 2B3, produced strong signals in the postexercise sample and moderate signals in the preexercise samples.

### Quantification of the titin fragment

**Fig 4:**
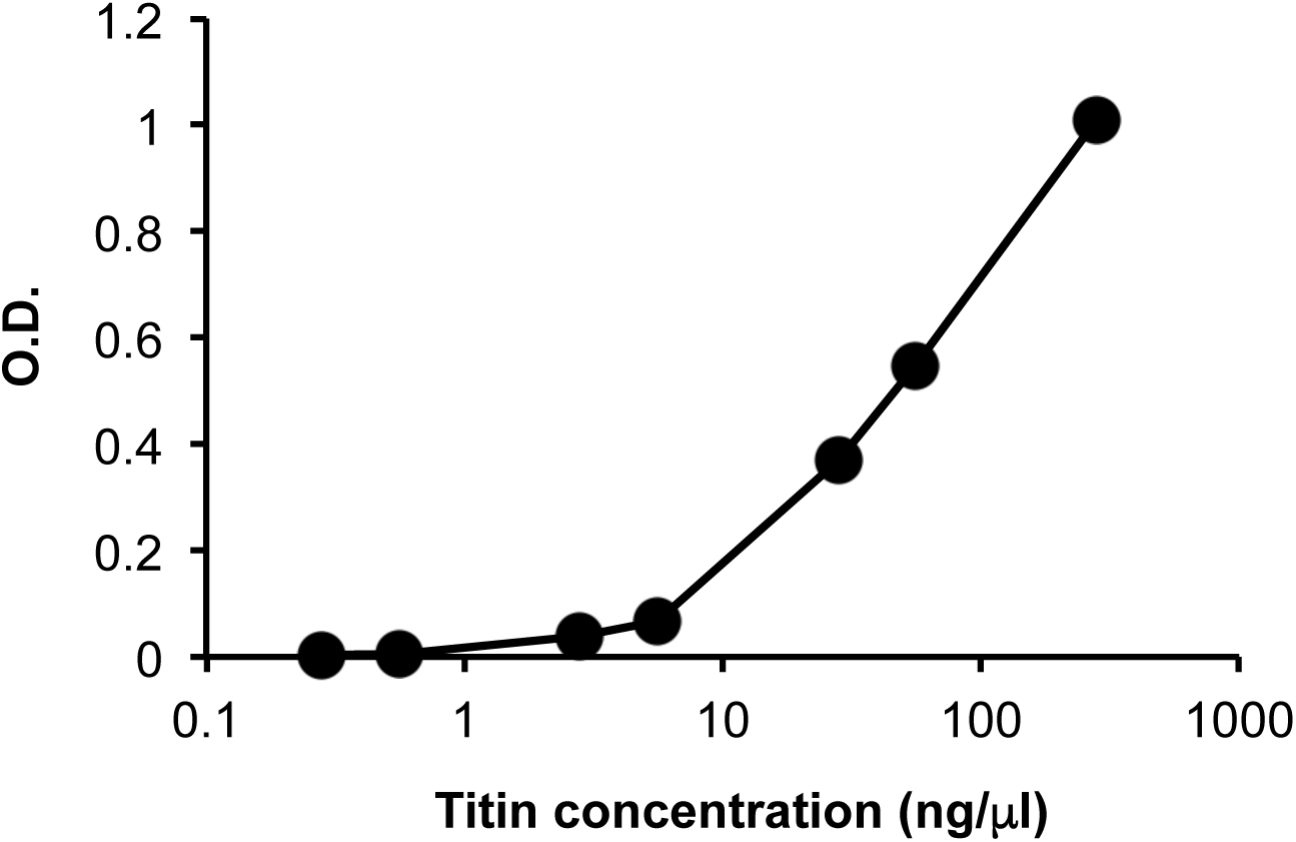
A typical standard curve for the measurement of the N-terminal fragment of human titin. We established an ELISA to measure the concentration of the N-terminal fragment of human titin using anti-human titin monoclonal antibodies 7D3 and 2B3. The GST-tagged recombinant human titin (amino acids 1–111) was detected with the ELISA in a concentration-dependent manner.

**Fig 5:**
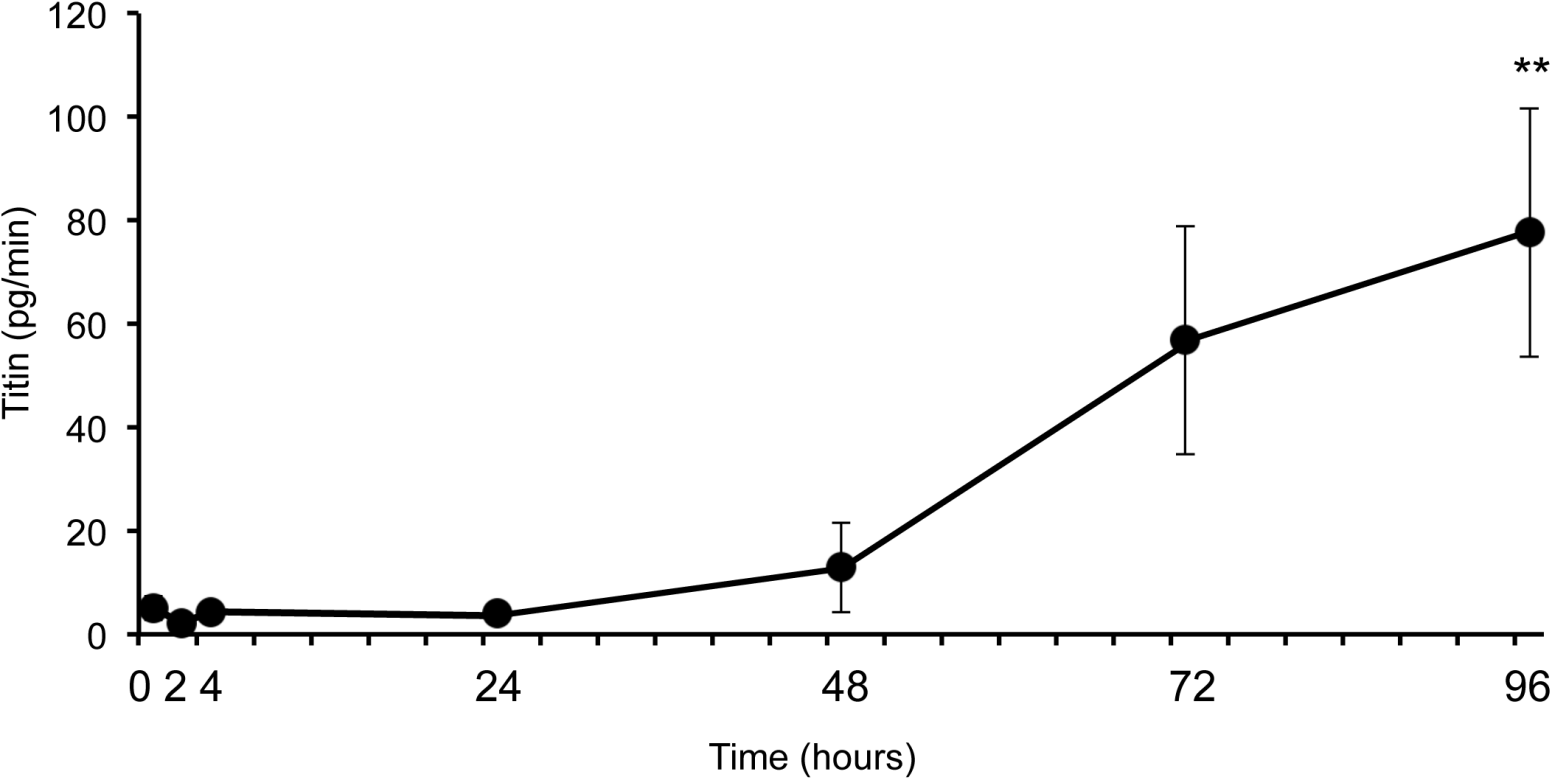
Rate of urinary titin fragment excretion after eccentric exercise. The urinary titin fragment excretion rate was calculated as the concentration × gross amount of urine per minute. The urinary titin fragment excretion rate increased significantly 96 h after exercise. Values are expressed as means ± SE (n = 9). **p < 0.01 vs Pre.

We established an ELISA to measure the urinary titin fragment. Based on an immunoblotting analysis, we performed the ELISA with monoclonal antibodies 7D3 and 2B3. To confirm the specificity of the detection, their reactivity to the recombinant N-terminal titin fragment (amino acids 1–111) was measured (Fig 4). We then measured the levels of the titin fragment in the urine of nine subjects before and after eccentric exercise. The titin fragment excretion rates were 5.1 ± 2.3 pg/min (Pre), 2.2 ± 0.6 pg/min (2 h), 4.2 ± 1.5 pg/min (4 h), 4.0 ± 1.4 pg/min (24 h), 12.9 ± 8.6 pg/min (48 h), 56.9 ± 22.0 pg/min (72 h), and 77.6 ± 23.0 pg/min (96 h), respectively. As shown in Fig 5, the urinary titin fragment excretion rate increased significantly 96 h after exercise (p < 0.01).

### Relationships between the excretion rates of the urinary titin fragment and blood markers of muscle damage

As shown in Table 2, after eccentric exercise, there were positive correlations between the percentage change in the excretion rates of the titin fragment and CK at 48 h after eccentric exercise (r = 0.98, p < 0.01), the titin fragment and CK at 48 h and 72 h (r = 0.79, p < 0.05 and r = 0.81, p < 0.01, respectively), the titin fragment and Mb at 48 h (r = 0.98, p < 0.01), the titin fragment and Mb at 72 h (r = 0.67, p < 0.05), the titin fragment at 48 h and Mb at 72 h (r = 0.88, p < 0.01), the titin fragment at 48 h and LDH at 72 h (r = 0.91, p < 0.01), the titin fragment and ALD at 48 h (r = 0.98, p < 0.01), and the titin fragment at 48 h and ALD at 72 h (r = 0.94, p < 0.01).

**Table 2:** Pearson's correlation coeffcient matrix of titin and muscle damage markers.

	titin 48h	titin 72h	CK 48h	CK 72h	Mb 48h	Mb 72h	LDH 48h	LDH 72h	ALD 48h	ALD 72h
titin 48h		0.39	0.98**	0.79*	0.98**	0.88**	0.57	0.91**	0.98**	0.94**
titin 72h	0.39		0.36	0.81	0.28	0.67	0.01	0.63	0.33	0.60
CK 48h	0.98**	0.36		0.80	0.99	0.88	0.62	0.93	1.00	0.95
CK 72h	0.79*	0.81**	0.80		0.74	0.96	0.36	0.94	0.78	0.94
Mb 48h	0.98**	0.28	0.99	0.74		0.83	0.61	0.89	0.99	0.92
Mb 72h	0.88**	0.67*	0.88	0.96	0.83		0.55	0.97	0.87	0.95
LDH 48h	0.57	0.01	0.62	0.36	0.61	0.55		0.56	0.67	0.47
LDH 72h	0.91**	0.63	0.93	0.94	0.89	0.97	0.56		0.92	0.97
ALD 48h	0.98**	0.33	1.00	0.78	0.99	0.87	0.67	0.92		0.94
ALD 72h	0.94**	0.60	0.95	0.94	0.92	0.95	0.47	0.97	0.94	

Creatine kinase (CK), myoglobin (Mb), lactate dehydrogenase (LDH), aldolase (ALD), 48 hour post-exercise (48 h), and 72 hour post-exercise (72 h). All data are calculated as percent changes for the pre-exercise values.

* p<0.05

** p<0.01.

### Relationships between the urinary titin fragment excretion rate, creatine kinase, myoglobin, and muscle symptoms

As shown in Table 3, after eccentric exercise, there were positive correlations between the percentage changes in the titin fragment at 48 h and DOMS at 48 h and 72 h (r = 0.91, p < 0.01 and r = 0.78, p < 0.05, respectively). There were negative correlations between the percentage changes in the excretion rates of the titin fragment at 72 h and ROM 48 h and 72 h (r = -0.71, p < 0.05 and r = -0.76, p < 0.05, respectively), the titin fragment at 72 h and torque post exercise (r = -0.72, p < 0.05).

**Table 3:** Pearson's correlation coefficient matrix of titin, muscle damage markers, and muscle symptoms.

	titin 48h	titin 72h	CK 48h	CK 72h	Mb 48h	Mb 72h	DOMS 48h	DOMS 72h	ROM 48h	ROM 72h	torque post	torque 24h
titin 48h		0.39	0.98	0.79	0.98	0.88	0.91 **	0.78 *	-0.01	-0.12	-0.59	-0.09
titin 72h	0.39		0.36	0.81	0.28	0.67	0.16	0.11	-0.71 *	-0.76 *	-0.72 *	0.26
CK 48h	0.98	0.36		0.80	0.99	0.88	0.93 **	0.80 *	0.03	-0.10	-0.51	-0.05
CK 72h	0.79	0.81	0.80		0.74	0.96	0.67	0.59	-0.34	-0.43	-0.74 *	0.18
Mb 48h	0.98	0.28	0.99	0.74		0.83	0.94 **	0.82 *	0.08	-0.04	-0.48	-0.07
Mb 72h	0.88	0.67	0.88	0.96	0.83		0.72 *	0.59	-0.10	-0.24	-0.75 *	-0.07
DOMS 48h	0.91 **	0.16	0.93 **	0.67	0.94 **	0.72 *		0.96	0.03	0.03	-0.52	0.23
DOMS 72h	0.78 *	0.11	0.80 *	0.59	0.82 *	0.59	0.96		0.02	0.12	-0.46	0.35
ROM 48h	-0.01	-0.71 *	0.03	-0.34	0.08	-0.10	0.03	0.02		0.89	0.42	-0.75
ROM 72h	-0.12	-0.76 *	-0.10	-0.43	-0.04	-0.24	0.03	0.12	0.89		0.43	-0.54
torque post	-0.59	-0.72 *	-0.51	-0.74 *	-0.48	-0.75 *	-0.52	-0.46	0.42	0.43		0.10
torque 24h	-0.09	0.26	-0.05	0.18	-0.07	-0.07	0.23	0.35	-0.75	-0.54	0.10	

Creatine kinase (CK), Myoglobin (Mb), delayed-onset muscle soreness (DOMS), range of motion (ROM), post-exercise (post), 24 hour post-exercise (24h), 48 hour post-exercise (48h), and 72 hour post-exercise (72h). All data are calculated as percent changes for the pre-exercise values.

* p<0.05

** p<0.01.

CK activities at 48 h were correlated with DOMS at 48 h and 72 h (r = 0.93, p < 0.01 and r = 0.80, p < 0.05, respectively). There was a negative correlation between the percentage changes in the CK activity at 72 h and torque post exercise (r = -0.74, p < 0.05).

Mb at 48 h was correlated with DOMS at 48 h and 72 h (r = 0.94, p < 0.01 and r = 0.82, p < 0.05, respectively). Mb at 72 h was correlated with DOMS at 48 h (r = 0.72, p < 0.05). There was a negative correlation between the percentage changes in Mb at 72 h and torque post exercise (r = -0.75, p < 0.05).

In this study, using a proteomic analysis, we detected the N-terminal fragment of the titin molecule in urine collected after acute eccentric exercise. We then verified the presence of the titin fragment with immunoblotting and quantified the concentrations before and after eccentric exercise. The urinary titin fragment increased significantly 96 h after eccentric exercise, and the changes in its concentration were strongly correlated with changes in both muscle symptoms and blood biomarkers of muscle damage observed previously [9, 18].

To our knowledge, this is the first report to show that a titin fragment can be detected in the urine. Titin is the largest protein in humans, with a molecular weight up to 3,700 kDa, and is known to be a structural sarcomere protein of striated muscle [25, 26]. The I-band region of titin underlies myofibril elasticity, and links the Z-line to the M-line, keeping the A-line in the center of the sarcomere, giving the appearance of cross-striations in skeletal and cardiac muscles. Titin filaments overlap the N-terminal ends of the Z-lines. The C-terminal titin regions from adjacent half-sarcomeres also overlap in the M-line [6, 27]. Because titin plays an important role in the passive and active contractility of skeletal muscle, its loss contributes to muscle weakness in titin-associated skeletal muscle diseases, such as spasticity and disuse atrophy [28].

In the present study, muscle strength decreased significantly until 24 h after exercise, but recovered on and after 48 h after exercise as compared with the preexercise value. On the other hand, muscle damage markers such as CK and Mb together with DOMS increased on and after 48 h after exercise. These results were similar with the previous studies (Horita et al., 1999, Vassilios paschalis et al., 2005).

The urinary excretion rates of the titin fragment were significantly correlated with muscle damage symptoms, including DOMS, reduced muscle strength and ROM. On the other hand, existing muscle damage markers such as CK and Mb were correlated with DOMS and reduced muscle strength but ROM. These results may suggest that the eccentric exercise-induced increase in urinary titin excretion rate associates with the reduction in ROM. We also demonstrated significant correlations between the changes in the titin fragment concentrations and the changes in serum CK, Mb, LDH, and ALD after acute eccentric exercise. These results suggest that the urinary titin fragment has potential utility as a non-invasive biomarker of muscle damage.

A number of titin isoforms are produced in different striated muscle tissues as the result of alternative splicing [29], and the titin isoforms are expressed differentially in cardiac and skeletal muscles [30, 31]: N2A (up to 3,700 kDa) in skeletal muscle [6], and N2B (3,000 kDa) and N2BA (3,300 kDa) in cardiac muscle [27]. The titin fragment detected in this study was estimated to be about 200 amino acids long, extending from the N-terminus of the molecule, based on its amino acid sequence and molecular weight (Figs 2 and 3B). Because the amino acid sequences of the titin isoforms do not differ in their N-terminal regions, we cannot exclude the possibility that this fragment was derived from the cardiac muscle, although it is very likely that the fragment was released from damaged skeletal muscle.

The mechanism underlying the urinary excretion of the titin fragment is currently unknown. During urine formation, glomerular filtration allows the passage of water and solutes into the urinary space, whereas the plasma proteins are retained. It is possible that the N-terminal fragment of titin is excreted into the urine after the fragmentation of the titin molecule in damaged muscle, because the size of the molecule is reduced. The delayed increase in titin fragment (96 h after eccentric exercise) may reflect complicated urinary excretion of the titin fragment. There are some papers published regarding titin cleavage by proteolytic enzymes such as calpain [32], however there is no report available for the N-terminal fragment of titin detected in the present study. Further research is required to clarify the mechanisms of titin cleavage and the urinary excretion of the titin fragment.

In conclusion, the urinary titin fragment identified here is potentially a noninvasive biomarker of muscle damage because it reflects the changes that occur in the serum markers of muscle damage and in the muscle symptoms observed after exercise-induced muscle damage.

## Supporting information

S1 Table A list of 99 proteins differentially expressed before and after acute eccentric exercise, detected with 2D-DIGE. Molecular weight (Mw) is the theoretical value for the respective protein.
(XLSX)

## Abbreviations

2D-DIGE: two-dimensional differential in-gel electrophoresis
ACN: acetonitrile
ALD: aldolase
BSA: bovine serum albumin
CK: creatine kinase
DOMS: delayed-onset muscle soreness
ELISA: enzyme-linked immunosorbent assay
HPLC: high-performance liquid chromatography
HRP: horseradish peroxidase
IPG: immobilized pH gradient
LC-MS/MS: liquid chromatography-tandem mass spectrometry
LDH: lactate dehydrogenase
PAGE: polyacrylamide gel electrophoresis
PBS: phosphate-buffered saline
ROM: range of motion
SDS: sodium dodecyl sulfate
VAS: visual analogue scale
